# CRMP2 derived from cancer associated fibroblasts facilitates progression of ovarian cancer via HIF-1α-glycolysis signaling pathway

**DOI:** 10.1038/s41419-022-05129-5

**Published:** 2022-08-04

**Authors:** Yunfeng Jin, Saiyan Bian, Hui Wang, Jiahang Mo, He Fei, Li Li, Tong Chen, Hua Jiang

**Affiliations:** 1 Department of Gynecology, Obstetrics and Gynecology Hospital, Fudan University, Shanghai, 200011 China; 2 Department of Obstetrics and Gynecology, Affiliated Hospital of Nantong University, Nantong, Jiangsu 226001 China; 3 Research Center of Clinical Medicine, Affiliated Hospital of Nantong University, Nantong, Jiangsu 226001 China; 4 Department of Hematology, Huashan Hospital, Fudan University, Shanghai, 200040 China

**Keywords:** Cancer, Tumour biomarkers

## Abstract

As the predominant stroma cells of tumor microenvironment (TME), cancer associated fibroblasts (CAFs) are robust tumor player of different malignancies. However, less is known about the regulatory mechanism of CAFs on promoting progression of ovarian cancer (OvCA). In the present study, the conditioned medium of primary CAFs (CAF-CM) from OvCA was used to culture cell lines of epithelial ovarian cancer (EOC), and showed a potent role in promoting proliferation, migration and invasion of cancer cells. Mass spectrum (MS) analysis identified that Collapsin response mediator protein-2 (CRMP2), a microtubule-associated protein involved in diverse malignancies, derived from CAFs was a key regulator responsible for mediating these cell events of OvCA. In vitro study using recombinant CRMP2 (r-CRMP2) revealed that the protein promoted proliferation, invasion, and migration of OvCA cells through activation of hypoxia-inducible factor (HIF)-1α-glycolysis signaling pathway. The CRMP2 was abundantly expressed in OvCA, with a well correlation with metastasis and poor prognosis, as analyzed from 118 patients’ samples. Inhibition of the CRMP2 derived from CAFs by neutralizing antibodies significantly attenuated the tumor size, weights, and metastatic foci numbers of mice in vivo. Our finding has provided a novel therapeutic clue for OvCA based on TME.

## Introduction

Ovarian cancer (OvCA) is a leading fatal gynecologic malignancy worldwide [1]. Despite traditional debulking surgery combined with chemotherapy and adjuvant therapies, 75% of patients develop advanced-stage (III–IV) with a low 5-year survival rate and recurrence within 3 years [2, 3]. Approximately 90% of cases are of epithelial ovarian cancer (EOC) characterized by dissemination and metastasis [4]. Therefore, the metastatic and recurrence mechanisms of OvCA need to be profoundly elucidated.

Recently, growing evidence suggested that cancers are not solely considered as core neoplastic cells, but as a dynamic crosstalk with the milieu of the tumors. By establishing communication with the tumor microenvironment (TME), tumor cells gain potent ability to sustain growth and metastasis [5–7]. Cancer-associated fibroblasts (CAFs), the predominant stromal cells of TME, modulate tumor progression by secreting various pro-inflammatory cytokines (IL-6 [8], IL-8 [8], TGF-β [9], hepatocyte growth factor (HGF) [10], vascular cell adhesion molecules-1 (VCAM-1) [11], and chemokines (CXCL12, CXCL14, CXCL1) [12–14]. Cancer cells are a reservoir of different soluble factors which can recruit and activate normal fibroblasts and reprogram them to produce various factors that favor cancer growth and spread [15, 16]. As a feedback, cancer cells can activate CAFs to produce various tumor-associated mediators to favor cancer growth and spread. Given the CAFs are involved in progression of OvCA in cross-talk with various signaling pathways, the extensive insights into the regulatory mechanisms of CAFs on the development of OvCA are greatly significant.

In the present study, we found that Collapsin response mediator protein-2 (CRMP2), also known as dihydropyrimidinase-like protein-2 (DPYSL2), was abundant in the supernatant of CAFs in comparison with normal ovarian fibroblasts (NOFs) by mass spectrum (MS) analysis. CRMP2, a 62KDa microtubule-associated protein (MAP), has been shown to participate in progression of multiple types of tumors [17–21]. We herein demonstrated that CRMP2 derived from CAFs promoted tumor progression in vivo and in vitro. Unbiased RNA-seq analysis revealed that CRMP2 activated hypoxia inducible factor (HIF)-1α-glycolysis signaling pathway.

By using the “Warburg effect”, cancer cells exhibit atypical glucose metabolism features [22] to mediate tumor progression [23, 24]. We also demonstrated that CRMP2 significantly promoted aerobic glycolysis of cancer cells. Tumor microarray (TMA) analysis showed that CRMP2 was correlated with metastasis and poor prognosis. Taken together, our results revealed the mechanism of CAF-derived CRMP2 in facilitating the progression of OvCA.

## Results

### CAFs promoted proliferation, migration and invasion of ovarian cancer cells (OCCs)

**Fig. 1:**
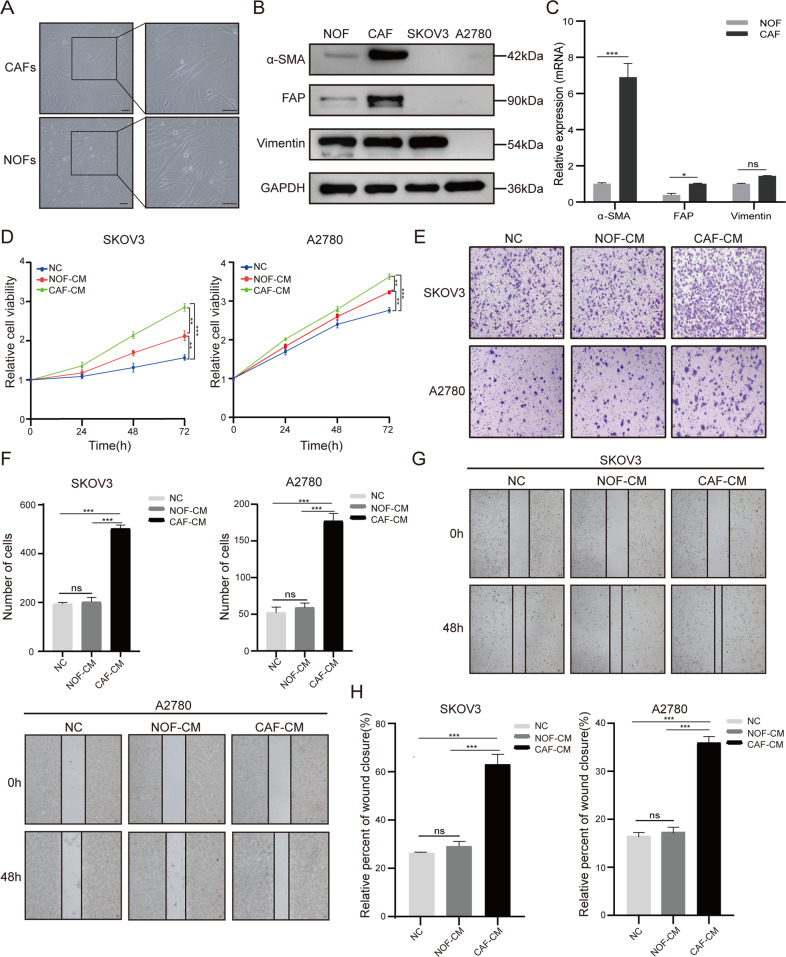
The conditioned medium (CM) of CAFs promotes proliferation, migration, and invasion of OCCs. **A** The typical morphological images of primary CAFs and normal ovarian fibroblasts (NOFs). Bar = 100 μm. **B**, **C** Western blotting and RT-qPCR analysis presented the expression of their representative markers (α-SMA, FAP, and vimentin). **D** CCK8 assay was used to analyze the viability of SKOV3 and A2780 cells co-cultured with CAF-CM and NOF-CM. **E**, **F** Cell invasion was assessed by Transwell assay in SKOV3 and A2780 cells after 48-h co-cultured with conditioned medium (100×magnification). **G**, **H** Cell migration ability was measured by wound healing assay in SKOV3 and A2780 cells (100×magnification). The quantitative analyses were performed using ImageJ software. Results are presented as the mean ± SD of three independent experiments. *P < 0.05, ***P < 0.001, ns not significant.

Primary CAFs and NOFs were isolated from EOC patients, as previously described [25]. They showed spindle- and stellate-like morphology with expression of specific markers α-SMA, FAP, and vimentin (Fig. 1A). Western blotting, RT-qPCR and immunofluorescence analysis demonstrated that α-SMA and FAP were both significantly increased in CAFs comparing with those in NOFs, while vimentin was unaffected (Fig. 1B, C and Supplementary Fig. 1A).

CAFs play diverse supportive roles in different malignancies [26–29]. To elucidate their roles in OvCA, the conditioned medium of CAFs (CAF-CM) was used to culture EOC cell lines (SKOV3 and A2780). CCK8 assay revealed that both CAF-CM and NOF-CM were able to promote OCCs proliferation (*P* < 0.01) with higher efficiency for the CAF-CM (*P* < 0.001) (Fig. 1D). Transwell assay showed that CAF-CM remarkably facilitated invasion of both cell lines in comparison with NOF-CM or negative control (NC) (Fig. 1E, F). Wound healing assay displayed that both SKOV3 and A2780 cells cultured with CAF-CM for 48 h exhibited increased migration ability (Fig. 1G, H). These results indicate that CAFs can promote progression of OvCA.

### Differential expressed proteins were characterized from CAF-CM and NOF-CM

**Fig. 2:**
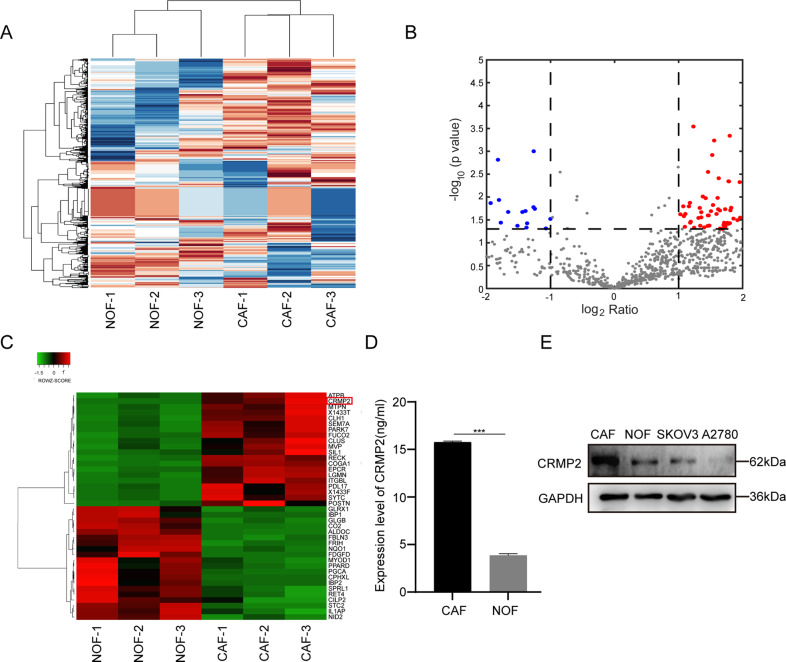
Differential expressed proteins were characterized from CAF-CM and NOF-CM. **A** Mass spectrum analysis of significant differential proteins in the supernatant of CAFs and NOFs. **B** Volcano plot depicted 95 differential proteins which were abundant in CAFs (red spots, log2 ratio > 2, *P* < 0.05). The bule spots represented the down-regulated proteins. **C** The top 20 significantly differentiated proteins were listed in the heatmap. CRMP2 (DPYL2, DPYSL2) was marked with a red square frame. **D** ELISA assay detected the expression of CRMP2 in the supernatant of CAFs and NOFs. **E** Western blotting analyzed the expression of CRMP2 in CAFs, NOFs, SKOV3, and A2780 cells. Results are presented as the mean ± SD of three independent experiments. ***P < 0.001.

To further take an insight into different biological behaviors caused by CAFs and NOFs, a 48-h conditional supernatant was collected for the MS analysis. As data showed, a total of 126 differential proteins were expressed in CAF-CM (Fig. 2A), among which 95 (red spots) and 31 (blue spots) were obviously upregulated and downregulated in CAF-CM, respectively (Fig. 2B). Most of the differential proteins were found to distribute in extracellular exosome (Supplementary Fig. 1B). The top ten biological function processes, molecular functions and KEGG metabolic pathways were presented in Supplementary Fig. 1C–E, and the most 20 differential proteins were shown in Fig. 2C. Eight differentially upregulated proteins, including ITGBL1, DPYSL2 (CRMP2), COGA1, SYTC, POSTN, 1433 F, LGMN, and PARK7, were significantly abundant in CAF-CM (log2 ratio > 3.00, *P* < 0.05) (Supplementary Table 1). As CRMP2 has been shown to participate in various malignancies and was enriched in differential proteins of CAF-CM (log2 ratio = 5.44, *P* < 0.007), it was possibly the core regulator of CAFs in promoting progression of OvCA. ELISA assay was performed to measure the contents of CRMP2 in CAF-CM, showing consistent results as those of MS analysis (Fig. 2D). Immunoblot indicated that CRMP2 was upregulated in CAFs, comparing to the NOFs, SKOV3, and A2780 cells (Fig. 2E).

### CRMP2 derived from CAFs promoted proliferation, migration and invasion of OCCs

**Fig. 3:**
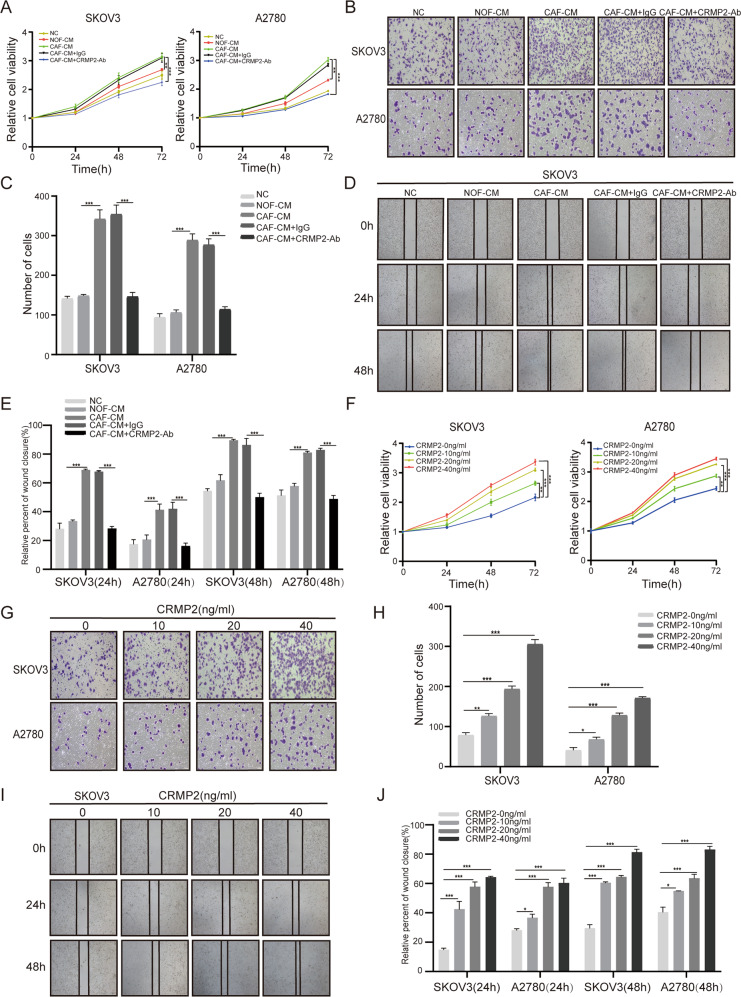
CRMP2 derived from CAFs promotes proliferation, migration, and invasion of OCCs. **A**–**E** SKOV3 and A2780 cells were cultured with CAF-CM, NOF-CM, CAF-CM + IgG, and CAF-CM + CRMP2 neutralizing antibody (Ab) (5 ng/ml). **A** CCK8 assay detected the cell viability of SKOV3 and A2780 cells after neutralizing CRMP2 of CAF-CM. **B**, **C** Cell invasion ability was validated by Transwell assay after 48 h (100×magnification). **D**, **E** Cell migration was measured via wound healing assay in the time point of 24 h and 48 h after blocking CRMP2 of CAF-CM, respectively (40×magnification). **F**-**J** SKOV3 and A2780 cells were treated with different dose of human recombinant CRMP2(r-CRMP2). **F** CCK8 assay was performed to examine OCCs viability. **G**, **H** Transwell assay was used to estimate cell invasion ability in different concentration of r-CRMP2 (100×magnification). **I**, **J** Wound healing assay was used to measure cell migration ability after treating with r-CRMP2 at 24 h and 48 h (40×magnification). Tanswell and wound healing quantitative analyses were performed using ImageJ software. Results are presented as the mean ± SD of three independent experiments. *P < 0.05, **P < 0.01, ***P < 0.001, ns not significant.

To address the roles of CRMP2 derived from CAFs in OvCA, SKOV3 and A2780 cells were cultured with CAF-CM, NOF-CM, CAF-CM + IgG and CAF-CM + CRMP2 neutralizing antibody (CRMP2-Ab). ELISA assay showed that CRMP2 antibodies effectively neutralized CRMP2 in CAF-CM (Supplementary Fig. 2A). CCK8 assay demonstrated that OCCs co-cultured with CAF-CM were more viable than with NOF-CM. However, treatment with CRMP2-Ab significantly inhibited the proliferation of cells (*P* < 0.001) (Fig. 3A). Transwell and wound healing assay showed that both SKOV3 and A2780 cells co-cultured with CAF-CM displayed more invasive and migratory abilities, and addition of CRMP2-Ab efficiently mitigated these effects (Fig. 3B–E, and Supplementary Fig. 2B). Also, addition of r-CRMP2 into the culture medium was able to remarkably enhance proliferation, invasion and migration abilities of OCCs (Fig. 3F–J and Supplementary Fig. 2C).

It is interesting to understand the role of endogenous CRMP2 in the cell events of OvCA, as CRMP2 was detected in the SKOV3 and A2780 cells by RT-qPCR and immunoblot, showing a differential abundance (Supplementary Fig. 2D). Thus, CRMP2 in SKOV3 was knocked down with siRNA3 (KD3) and siRNA4 (KD4), while in A2780 cells was overexpressed with CRMP2-plasmids to examine the loss-of and gain-of functions (Supplementary Fig. 2E–H). Results revealed that silencing CRMP2 in SKOV3 cells inhibited proliferation, migration and invasion of the cells (Supplementary Fig. 3A–E), whereas overexpressing CRMP2 in A2780 cells resulted in more malignant properties (Supplementary Fig. 3F–J). Therefore, CRMP2 either derived from CAFs or endogenously expressed in OvCA can act as tumor players in OvCA progression.

### CRMP2 derived from CAFs activated HIF-1α-glycolysis signaling pathway

**Fig. 4:**
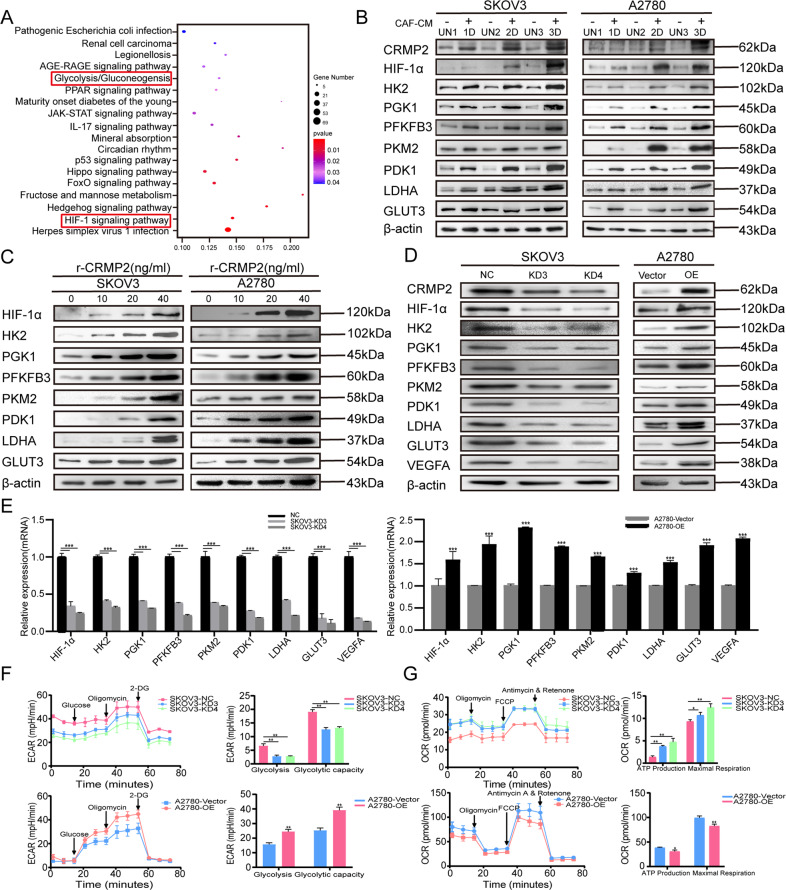
CRMP2 derived from CAFs activates HIF-1α-glycolysis signaling pathway. **A** RNA-sequencing analysis of the differential genes based on A2780 cells treated with r-CRMP2. KEGG bubble map displayed the top 18 pathways enriched in the differential genes (log2 (Fold Change) > 1 and Q-value < 0.05). HIF-1α signaling pathway and glycolysis/gluconeogenesis pathway were both statistically significant (*P* < 0.05). Size of bubble represented the gene number while the color represents the *P*-value. **B** Western blotting validated the HIF-1α-glycolysis pathway using co-culture models from one day (1D) to three days (3D). UN1-3: cells untreated with CAF-CM were set as the controls of 1–3 day. **C** Western blotting detected the HIF-1α-glycolysis pathway after treatment with different concentrations of r-CRMP2 (ng/ml). **D**, **E** Western blotting and RT-qPCR analysis of the HIF-1α-glycolysis pathway after knocking down CRMP2 in SKOV3 cells and overexpressing CRMP2 in A2780 cells. **F**, **G** Seahorse XF Extracellular Analysers were used to detect extracellular acidification rate (ECAR) and oxygen consumption rate (OCR) on CRMP2-silenced SKOV3 (SKOV3-KD) and CRMP2-overexpressed A2780 (A2780-OE) cells. **F** Respective images of ECAR measurement in SKOV3-KD and A2780-OE cells. Glycolysis and glycolytic capacity were analyzed in SKOV3-KD and A2780-OE cells. **G** Respective images of OCR measurement in SKOV3-KD and A2780-OE cells. ATP production and maximal respiration were analyzed using GraphPad software. Results are presented as the mean ± SD of three independent experiments. *P < 0.05, **P < 0.01, ***P < 0.001. UN untreated. NC negative control.

To investigate the possible signaling pathways involved in OvCA progression caused by CRMP2, we performed RNA-sequencing and bioinformatics analysis on A2780 cells treated with r-CRMP2 (A2780 + r-CRMP2). Data showed that a total of 1045 and 1500 genes were upregulated and downregulated in treated group compared with the control (log2 (Fold Change) > 1 and Q-value < 0.05), respectively (Supplementary Fig. 4A). KEGG pathway analysis revealed that the top three enriched pathways were involved in the herpes simplex virus infection, HIF-1 signaling and Hedgehog signaling (log2 (Fold Change) > 1 and Q-value < 0.05) (Fig. 4A). As what was analyzed from KEGG pathway mapper, the glycolysis pathway was regulated by the HIF-1α signaling pathway and involved in different malignancies [30–34]. Glycolysis signaling was contained in the most enriched 18 signaling pathways and 16 genes were enriched in the downstream of HIF-1 signaling, including *HK2, PFKFB3, PDK1, PGK1* and *VEGFA* (Fig. 4A). GO analysis revealed that the predominant biological process of the differential genes was mainly associated with hypoxia and glycolysis (GOBPID:00016666, *P* = 4.73e-06) (Supplementary Fig. 4B). In addition, CRMP2 was positively co-related with HIF-1α via correlation analysis (R = 0.46, *P*-value = 0) (Supplementary Fig. 4C).

Immunoblot and RT-qPCR were performed to substantiate HIF-1α-glycolysis pathway. As HIF-1 could be activated through the most classic signaling pathway PI3K-Akt-mTOR [35], we also validated the upstream pathway PI3K-Akt-mTOR using Immunoblot. SKOV3 and A2780 cells were either cocultured with CAFs or treated with 0–40 ng/ml r-CRMP2. Results showed that the protein levels of CRMP2, HIF-1α, glycolysis-related enzymes, GLUT3, P-Akt and P-S6k were significantly increased when cells co-cultured with CAFs for 1 day to 3 days (Fig. 4B, Supplementary Fig. 4D, E and Supplementary Fig. 5). They were also induced by r-CRMP2 in a dose-dependent manner (Fig. 4C, Supplementary Fig. 4F, G and Supplementary Fig. 5). To shed light on the endogenous roles of CRMP2 in regulation of glycolysis-related enzymes, gain the function in A2780 cells and loss of the function in SKOV3 cells for the CRMP2 were performed. Results displayed that silencing CRMP2 in SKOV3 cells significantly reduced the protein levels of HIF-1α and its downstream glycolysis-related enzymes, including *HK2, PGK1, PFKFB3, PKM2, PDK1* and *LDHA*. Meanwhile, the expression of GLUT3 and VEGFA were both downregulated. Contrarily, overexpression of CRMP2 in A2780 cells made the opposite effects (Fig. 4D, E). Noteworthily, CRMP2 derived from CAFs was shown to participate in angiogenesis by promoting production of VEGFA (Fig. 4D, E), and treatment with the CRMP2-Ab could effectively inhibit tubule formation of human umbilical vein endothelial cells (HUVECs) (Supplementary Fig. 6A), while r-CRMP2 was able to enhance tubule formation (Supplementary Fig. 6B).

Seahorse XF Extracellular Analysers were subsequently used to detect the extracellular acidification rate (ECAR) in CRMP2-silenced SKOV3 (SKOV3-KD) and CRMP2-overexpressed A2780 (A2780-OE) cells. ECAR was significantly decreased in CRMP2-silenced SKOV3 cells but increased in the A2780-OE group (Fig. 4F). oxygen consumption rate (OCR), which reflects mitochondrial respiration, displayed an opposite tendency to those of ECAR (Fig. 4G). The results indicate that CRMP2 is able to mediate hypoxia and glycolysis of OvCA *via* HIF-1α-glycolysis signaling pathway.

### CRMP2 derived from CAFs promoted tumor growth and metastases in vivo

**Fig. 5:**
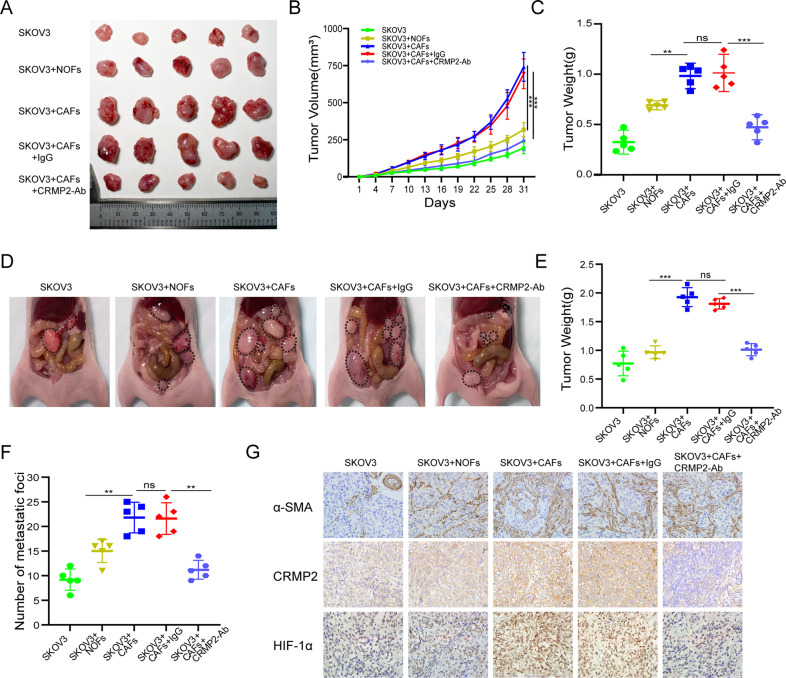
CRMP2 derived from CAFs promotes tumor growth and metastases in vivo. **A** The tumor xenografts were photographed on the 31st day of the experiment. IgG and neutralization antibody (Ab) were injected intraperitoneally on the second day of every three days. **B** Tumor volumes were recorded every three days. **C** Tumor weights were measured at the end of the experiment. **D** Intraperitoneal metastasis models were conducted. The peritoneal metastatic images were photographed at the end of the experiment, and the representative images of each group were displayed (black dotted circle). **E** Metastatic tumor weights were recorded. **F** The number of metastatic foci were calculated. **G** Immunohistochemistry (IHC) analysis of α-SMA, CRMP2 and HIF-1α in mice tumors of each group. **P < 0.01, ***P < 0.001, ns not significant.

Xenograft tumor and intraperitoneal metastasis models were established in vivo using BALB/c nude mice. SKOV3 cells and CAFs/NOFs were mixed at a ratio of 4:1 and injected subcutaneously and intraperitoneally. IgG and CRMP2-Ab were injected intraperitoneally from the second day. Xenograft tumor volumes in SKOV3 cells mixed with CAFs group were 1-2-fold larger than those with NOFs (*P* < 0.001) or SKOV3 groups (*P* < 0.001) (Fig. 5A, B). Interestingly, the group treated with CRMP2-Ab obviously attenuated tumor growth (Fig. 5A, B). Accordingly, the tumor of SKOV3 + CAFs group were much heavier than those of other groups, and application of CRMP2-Ab significantly decreased tumor weights (*P* < 0.001) (Fig. 5C). However, the weights of mice were unaffected (data not shown).

In intraperitoneal metastasis model (Fig. 5D–F), CAFs significantly promoted the metastasis of SKOV3 cells. Tumor weights of SKOV3 + CAFs group were significantly increased comparing with those of SKOV3 (*P* < 0.001) and SKOV3 + NOFs groups (*P* < 0.001). Similarly, administration of CRMP2-Ab remarkably decreased the tumor weights and number of metastatic foci. Immunohistochemistry (IHC) analysis showed that CAFs grew in parallel with SKOV3, and CRMP2-Ab effectively attenuated CRMP2 and HIF-1α expression (Fig. 5G). Taken together, the data indicate that CRMP2 derived from CAFs promotes tumor growth and metastasis.

### CRMP2 contributed to poor prognosis in OvCA patients

**Fig. 6:**
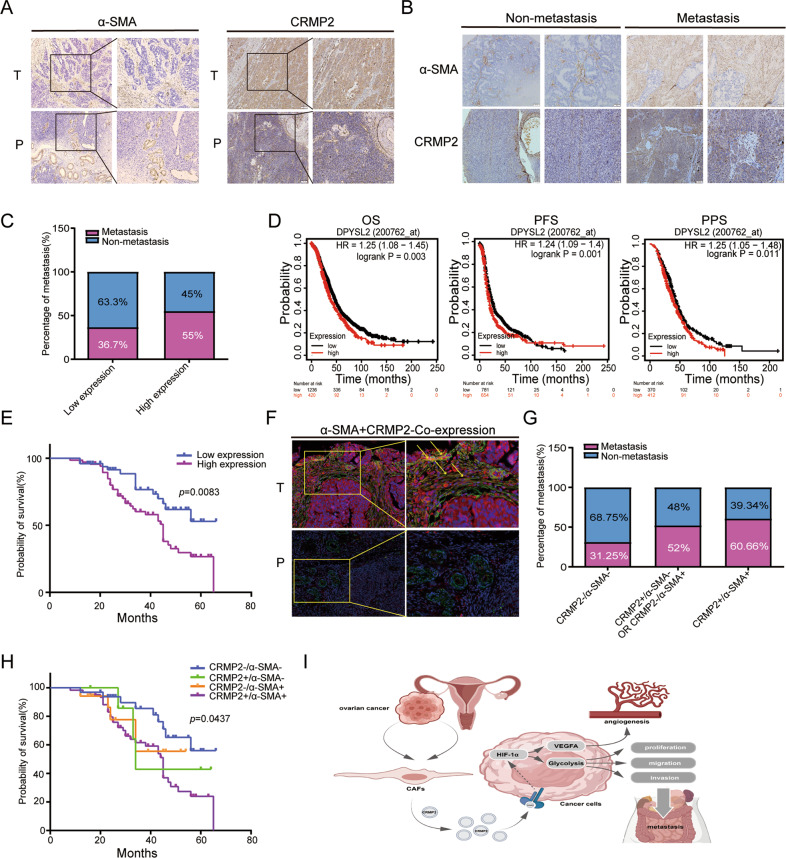
CRMP2 derived from CAFs contributes to poor prognosis in OvCA patients. **A** IHC analysis showed the expression of α-SMA and CRMP2 in epithelial ovarian cancer (EOC) tissues and para-carcinoma tissues (100×magnification, the zoomed-in section, 200×magnification). **B** Tissue microarray (TMA) containing 118 EOC patients was used to analyze expression of α-SMA and CRMP2 in non-and metastatic cases. **C** High expression of CRMP2 was correlated with tumor metastases. **D**, **E** Both Kaplan–Meier plotter and TMA analyses validated the high expression of CRMP2 that was related to poor prognosis. **F** There were α-SMA and CRMP2 co-expression in tumor stroma via immunofluorescence analysis (yellow arrow). No signal was detected in para-carcinoma tissues (200×magnification, the zoomed-in section, 400×magnification). Red and green fluorescence signal represented the expression of CRMP2 and α-SMA, respectively. While the yellow fluorescence signal represented co-expression. **G, H** CRMP2 + /α-SMA + represented the co-expression group, and the double positive group was associated with tumor metastases and probability of survival. **I** A schematic diagram showed that CRMP2 was secreted from CAFs and then stimulate the down-stream HIF-1α-glycolysis and HIF-1α-VEGFA signaling pathway to regulate the progression of ovarian cancer. OS overall survival, PPS post progression survival, PFS progression free survival.

We then performed IHC analysis and TAM assays to explore the correlation between CRMP2 and prognosis of OvCA patients. As shown in Fig. 6A, α-SMA was stained in tumor stroma and vascular smooth muscle in para-carcinoma tissues. CRMP2 was remarkably stained in the tumor tissues compared to the normal tissues. Immunofluorescence analysis showed the consistent results (Supplementary Fig. 6C, D).

TAM analysis revealed that α-SMA and CRMP2 were stained more abundantly in metastatic than in non-metastatic cases (*n* = 118) (Fig. 6B). CRMP2 was positively correlated with tumor metastasis and negatively correlated with the probability of survival (*P* = 0.0083) (Fig. 6C, E). Kaplan–Meier plot further validated the correlation of high expression of CRMP2 with poor overall survival, progression-free survival and post-progression survival (Fig. 6D). The clinical and pathological features of 118 cases and CRMP2 expression were presented in Table 1. CRMP2 expression was associated with histological stage (*P* = 0.004), FIGO stage (*P* = 0.012), peritoneal metastasis (*P* = 0.037), and lymphatic/peritoneal metastasis (*P* = 0.021). Immunofluorescence detected the co-localization of α-SMA and CRMP2 in tumor and para-carcinoma tissues, and they only co-expressed in tumor stroma tissues (yellow arrows). However, the co-expression signals were undetectable in normal tissues (Fig. 6F). In addition, the 118 patients were divided into four groups: CRMP2−/α-SMA−, CRMP2+/α-SMA−, CRMP2−/α-SMA+, and CRMP2+/α-SMA+. As shown in Fig. 6G, CRMP2+/α-SMA+ co-expression group accounted for 60.66% overall, and positively correlated with tumor metastases and an unsatisfactory prognosis (*P* = 0.0437) (Fig. 6H). The clinical and pathological parameters also demonstrated that CRMP2+/α-SMA+ co-expression was positively correlated with histological stage (*P* = 0.025), FIGO stage (*P* = 0.013), and lymphatic/peritoneal metastasis (*P* = 0.041) (Table 2). The results indicate that CRMP2 of CAFs in tumor stroma contributes to poor prognosis of OvCA patients.

**Table 1:** Clinicopathologic features and CRMP2 expression of TMA.

	CRMP2 expression			
Parameters	Total	Low	High	*P* value
Age (years)				
<50	42	19	23	0.339
≥50	76	30	46	
Histological Grade				
G1	22	15	7	
G2	14	8	6	0.004*
G3	82	26	56	
FIGO Stage				
I	52	26	26	
II	8	6	2	0.012*
III	58	17	41	
Lymphatic metastases				
No	77	36	41	0.083
Yes	41	13	28	
Peritoneal metastases				
No	62	31	31	0.037*
Yes	56	18	38	
Lymphatic & Peritoneal metastases				
No	58	30	28	0.021*
Yes	60	19	41	
Ascites				
No	74	33	41	0.248
Yes	44	16	28	
CA125 (U/ml)				
Normal (0–30)	30	16	14	0.096
High (>30)	88	33	55	

Chi-square test is used to analyze the statistical significance of CRMP2 expression in different subgroups. *P < 0.05.

Abbreviations: *CRMP2* collapsin response mediator protein-2, *FIGO* international federation of obstetrics and gynecology.

**Table 2:** Clinicopathologic features and CRMP2/α-SMA expression of TMA.

	CRMP2/α-SMA expression					
Parameters	Total	−/−	+/−	−/+	+/+	*P* value
Age (years)						
<50	42	12	3	6	21	0.992
≥50	76	20	5	11	40	
Histological Grade						
G1	22	11	1	4	6	
G2	14	5	2	3	4	0.025*
G3	82	16	5	10	51	
FIGO Stage						
I	52	19	5	6	22	
II	8	4	0	3	1	0.013*
III	58	9	3	8	38	
Lymphatic metastases						
No	77	25	6	11	35	0.227
Yes	41	7	2	6	26	
Peritoneal metastases						
No	62	23	5	7	27	0.053
Yes	56	9	3	10	34	
Lymphatic & Pertioneal metastases						
No	58	22	5	7	24	0.041*
Yes	60	10	3	10	37	
Ascites						
No	74	24	4	9	37	0.331
Yes	44	8	4	8	24	
CA125 (U/ml)						
Normal (0–30)	30	13	1	3	13	0.128
High (>30)	88	19	7	14	48	

Chi-square test is used to analyze the statistical significance of CRMP2/α-SMA expression in different subgroups. *P < 0.05.

Abbreviations: *CRMP2* collapsin response mediator protein-2, *α-SMA* α-smooth muscle actin, *FIGO* international federation of obstetrics and gynecology.

## Discussion

In the present study, MS analysis was performed to identify differential proteins in the supernatant of CAFs. The identified CRMP2 belongs to CRMP family (CRMP1-5), which are involved in the regulation of microtubule polymerization and axonal outgrowth [36]. CRMP2 mediates a range of other cellular functions, including apoptosis, proliferation, migration, and differentiation [37, 38]. CRMP2 is also ubiquitously expressed in non-neuronal cells, such as fibroblasts [17], leukocytes [39] and neuroblastoma cells [40]. It is recognized as a new class of MAP and is notable in cytoskeleton remodeling and maintenance of microtubule stability [41]. Recently, investigators have demonstrated that extracellular application of CRMP2 increased cytoplasmic calcium and further influence cell biological properties [42]. While aberrant expression of CRMP2 and nuclear phosphorylated CRMP2 were implicated in various malignancies, with increased expression in colorectal carcinoma [43] and bladder cancer [21], and decreased expression in breast cancer [19]. However, the exact role of CRMP2 in OvCA remains elusive.

In our research, we found that r-CRMP2 could promote proliferation, invasion, and migration of OCCs in vitro, while CRMP2-Ab significantly inhibited tumor growth and metastases in vivo. Similar to previous research [21, 43], CRMP2 was abundantly expressed in tumor stromal compared to para-carcinoma tissues. Co-expression analysis verified that α-SMA and CRMP2 were co-localized in tumor stroma, suggesting that CAFs secrete CRMP2 in tumor stroma and trigger reciprocal crosstalk between CAFs and OCCs. Contrary to what Shimada demonstrated [19], both our clinical data and Kaplan–Merier plot indicated that CRMP2 was positively correlated with poor prognosis and metastasis. Clinicopathological analysis demonstrated that CRMP2 was associated with FIGO stage, histological grade, and lymphatic/peritoneal metastasis. These distinct outcomes from different malignancies may attribute to tumor heterogeneity.

RNA-seq analysis revealed that application of CRMP2 could upregulate the expression of HIF-1 and the downstream glycolysis-related enzymes (*HK2, BFKFB3, PDK1, PGK1*), as well as VEGFA for angiogenesis. Hypoxia is recognized as a hallmark of TME, and HIF-1 is a key transcriptional regulator of tumor metabolism in hypoxic [44]. Activation of HIF-1 triggers a panel of down-stream genes (*GLUT1, PGK1, HK2, PGM, LDHA* and *MCT4*), and mediates tumor proliferation, angiogenesis, metastasis, cell invasion, migration and glucose metabolism [45, 46], showing consistency with our results. As a subunit of HIF-1, upregulation of HIF-1α has been reported in various human cancers with unsatisfactory outcomes [47–49]. We postulated that CRMP2 might regulate tumor progression *via* HIF-1α-glycolysis signaling pathway which has been found in several human malignances [24, 50]. Seahorse analysis validated that CRMP2 regulated glycolysis *via* enhancing the aerobic glycolytic capacity of tumor cells, while inhibiting their mitochondrial aerobic phosphorylation.

The question is raised as for how CRMP2 interacts with OCCs and then drives the HIF-1α-signaling pathway. As our MS analysis showed, the main subcellular localization of differential proteins was at extracellular exosomes. Findings support the note that CRMP2 could be released into extracellular milieu *via* exosomes and exerted its influence [36, 51]. Besides, Cecilia et al. found that in vitro CRMP2 increased cytoplasmic calcium through NMDA receptors (NMDARs) [42], and the NMDARs were also identified in SKOV3 and A2780 cells [52]. Calcium signaling pathway plays important role in cytoskeletal reorganization, cell migration and cancer metastasis [36], and regulates HIF-1 at different stages in different malignancies [53–55]. To our knowledge, HIF-1α is regulated *via* both O_2_-dependent and independent pathways [35]. HIF-1 activity requires its subunit HIF-1α, which is under the control of growth-factor signaling pathways, particularly the PI3K-Akt-mTOR pathway [56, 57]. However, the exact mechanisms have not been investigated in the present study, and they deserve further study.

In summary, we firstly showed that CRMP2 derived from CAFs was involved in progression of OvCA via the HIF-1α-glycolysis pathway. The identification of CRMP2 from CAFs provided us with a new understanding of CAFs in TME. Our results will be beneficial for the therapeutic strategies and provide a prognostic biomarker for diagnosis of OvCA.

## Material and methods

### Isolation of CAFs and NOFs

Primary CAFs and NOFs were isolated from fresh EOC tissues and their matched para-carcinoma tissues based on a previous study protocol [58] (See Supplementary Material and Methods). Cells were starved for 48 h, and conditioned media (CM) was obtained and stored at −80 °C. All experiments were based on 2–6 passages of fibroblasts, and data from each experiment were acquired from the same passage of CAFs and NOFs. This study was approved by the Institutional Ethics Committee of Fudan University. Informed consents were collected from all patients prior to analysis.

### Cell cultures and reagents

SKOV3 and A2780 cells were purchased from the American Type Culture Collection (ATCC, Manassas, VA, USA) and Shanghai Fuheng Biological Technology Co., respectively. SKOV3 cells were cultured in RPMI 1640 (Gibco, Gaithersburg, MD, USA) and A2780 was maintained in DMEM (Invitrogen Carlsbad, CA, USA) supplemented with 10% FBS. Primer cells and HUVECs were cultured in DMEM/F12 (HyClone, UT, USA) containing 10% FBS at 37 °C in a humidified 5% CO_2_ incubator.

### Western blotting analysis

Proteins were extracted and blocked with western blocking buffer (Beyotime Biotechnology, China) for 1 h at room temperature, and then incubated with primary antibodies overnight at 4 °C. Horseradish peroxidase (HRP)-conjugated secondary antibodies were then incubated for 2 h. Specific bands were visualized using an enhanced chemiluminescence (ECL) kit (Millipore, MA, USA). (See Supplementary Table 2 for the antibodies dilution rate).

### Quantitative real-time PCR (RT-qPCR)

Total RNA was extracted using RNA-Quick Purification kit (ES Science, Shanghai, China) and reverse transcribed using PrimeScript RT reagent kit (Takara, Japan). RT-qPCR analysis was conducted to quantitate mRNA relative expression using SYBR Premix Ex Taq (Takara) with β-actin as internal reference. Fluorescence signal was confirmed by melting curve analysis, and mRNA levels of target genes were calculated using the 2 ^–ΔΔCT^ method (See Supplementary Table 3 for the primers used).

### Cell viability assay

Cell viability was determined by cell counting-8 (CCK8) kit (Dojindo, Kumamoto, Japan) following the manufacturer’s instructions. SKOV3 and A2780 cells were seeded in 96-well plates (5 × 10^3^/well), respectively, and cultured at appropriate time points. Ten microliters of CCK8 reagent solution was added to each well and incubated for 2 h at 37 °C. Optical density value was measured at 450 nm wavelength.

### Cell invasion assay

Cell invasion assays were performed using 24-well plates with diluted BD Matrigel (1:8, BD, CA, USA) to pre-coat the transwell chambers (BD Biosciences, San Diego, CA, USA). Cell suspension containing 1 × 10^5^/100 μl was seeded on the pre-coated upper chambers. Different treated conditioned mediums were added into the lower chambers (500 μl/well). After incubating for 48 h, the unpenetrated cells and Matrigel on the upper chambers were removed. Cells were then fixed with 4% paraformaldehyde and stained with 0.1% crystal violet solution. Images were acquired from five random fields under a microscope. Each condition was set in triplicate.

### Wound healing assay

Cells were cultured in a 6-well plate with 80-100% confluence and were scratched with sterilized 200 μL pipette tip. Then cells were washed with 1 × PBS to remove suspended cells. The different group of conditioned mediums were replaced and cultured for 24 h and 48 h in a 37 °C incubator. Images were photographed by microcopy on the same point of wound marked on the plate.

### Liquid chromatography mass spectrum (LC-MS/MS) analysis

The LC-MS/MS analysis was performed as previously described [59]. Detailed procedures were presented in the supplementary materials and methods. MS data were operated in data-dependent acquisition mode to automatically switch between Orbitrap-MS and ion trap acquisition. MS spectra were acquired within 300 m/z to 1400 m/z range. Target ions already selected for MS/MS were dynamically excluded for 18 s, with 5000 minimum intensities. The LC-MS/MS data were analyzed using MaxQuant software version 1.6.0.16 (Max Planck Institute of Biochemistry, Germany).

### Enzyme-linked immunosorbent assay (ELISA)

CRMP2 levels in the supernatant of disparate groups were detected using human CRMP2 ELISA kit (LifeSpan Bioscience, Seattle, WA, USA) according to the manufacturer’s instructions. The absorbance intensity of each sample was detected at 450 nm wavelength using an automatic microplate reader (BioTek Instruments, Winooski, VT, USA).

### RNA sequencing and bioinformatics analysis

RNA sequencing was performed at Guangzhou RiboBio Co. Ltd. as previously described [60, 61]. The RNA samples included 6 replicates (3 for A2780-untreated and 3 for A2780-treated with r-CRMP2). A total of 51711457 reads generated by Illumina based on polyadenylated selected (Poly-A+) RNAs. Detailed protocol and bioinformatics analysis were provided in supplementary materials and methods.

### Cell transfection

Transient transfection was performed using Lipofectamine 3000 reagent (Invitrogen). SKOV3 cells were transfected with small interfering RNA (siRNA; Transheep, Shanghai, China) to knockdown CRMP2. Human DPYSL2 (CRMP2)-pTSB02-GFP-PURO plasmid (Transheep) was used to overexpress CRMP2 in OCCs. The siRNA sequences were listed in Supplementary Table 4.

### Animal experiments

The animal experiments included six-week-old female nude mice (BALB/c-nu) with five mice per group and were performed as previously [11]. The mixed cell suspension (2 × 10^6^ SKOV3 and 5 × 10^5^ CAFs/NOFs, 4:1) were injected subcutaneously or intraperitoneally. The CRMP2 neutralizing antibody or IgG (50 mg/kg, Abcam, Cambridge, MA, USA) was sequentially injected intraperitoneally from the second day. Tumor formation was recorded every 3 days, and tumor volume was calculated as followed: Tumor volume (mm^3^) = d^2^ × D/2, where D and d represents the tumor longest and shortest diameters. The animals were sacrificed and dissected at the appropriate time. All animal experiments followed the norms of the *Medical Research Animal Ethics Committee of Fudan University*.

### Immunohistochemistry (IHC) and tissue immunofluorescence

The 4 mm paraffin-embedded tissue sections were stained using IHC. EDTA buffer (Invitrogen) was used to perform heat-induced antigen retrieval at 100 °C. Endogenous peroxidase was eliminated using 3% H_2_O_2_. Tissues were blocked with 10% FBS and incubated with corresponding primary antibody overnight at 4 °C. Slides were then incubated with HRP Rabbit/mouse polymer before visualization with liquid diaminobenzidine tetrahydrochloride plus substrate DAB chromogen from Dako REAL ENvison (Capinteria, CA, USA). All slides were counterstained with haematoxylin. The target proteins’ expression was analyzed with ImageJ software (Rawak Software, Germany).

For tissue immunofluorescence, the IHC procedure were performed first. On the second day, after incubating with primary antibodies, tissue sections were incubated with Alexa Fluor 488 donkey anti-mouse IgG(H + L) and Alexa Fluor 594 donkey anti-rabbit IgG(H + L) (Life Technologies, Gaithersburg, MD, USA) secondary antibodies, respectively, or with mixed antibody solution. Cell nuclei were stained with 4’,6-Diamidino-2-phenylindole (DAPI). Immuno-stained slides were cover-mounted with Prolong Gold antifade reagent (Invitrogen).

### Measurement of extracellular acidification rate (ECAR) and oxygen consumption rate (OCR)

Seahorse XFe96 Extracellular Flux Analyser (Agilent Technologies, Billerica, MA, USA) was used to detect glycolytic capacity and cellular mitochondrial function. Measurements were performed using Seahorse Glycolysis and XF cell Mito Stress Test kits (Seahorse Bioscience, Billerica, MA, USA). Cells were seeded in an Agilent seahorse XF96 cell culture plate at a density of 4 × 10^4^ cells per well and cultured with prepared XF medium (seahorse, 102353-100) the day before determination. The base medium, DMEM, was supplemented with 10 mM glucose, 2 mM glutamine and 2 mM pyruvate. The cell plate was placed in a non-CO_2_ incubator for 1 h prior to the assay. After monitoring baseline respiration, for ECAR measurement, 10 mM glucose, 2 μM oligomycin and 50 μM 2-DG were automatically injected into each well. In order, 2 μM oligomycin, 1 μM FCCP and 0.5 μM rotenone/antimycin were used to measure OCR. OCR and ECAR values were analyzed using Wave 2.6 software (Agilent Technologies) after the number of cells was renormalized.

### Tissue microarrays (TMAs) analysis

CRMP2 and α-SMA expression in OvCA tissues was detected using TMAs obtained from the Clinic Pathology Centre of the Affiliated Hospital of Nantong University (Jiangsu Province, China). A TMA set consisted of two sample slides containing 119 EOC patients with one patient losing follow-up (*n* = 118). IHC intensity and positive frequency were analyzed using ImageJ software. Each sample was evaluated by two experienced pathologists. A composite scorning [62], the IHC intensity scores, defined as 0 (negative), 1 (weak), 2 (moderate), and 3 (strong), was used for statistical analysis. Positive frequency was scored as follows: 0 (0%), 1 (1–10%), 2 (11–50%), 3 (51–80%), and 4 (≥81%). The composite expression score (CES) was calculated as follows: CES = 4 × (intensity − 1) + frequency. The full CES range was from 0 to 12, with CES < 4 and 4–12 defined as low and high expression case, respectively.

### Statistical analysis

All the data were expressed as the mean ± SD. Significant differences were analyzed by Student’s t-test, and one-way or two-way ANOVA. Multivariate Cox regression models were used to analyze the correlations between risk factors and survival outcomes. Statistical analysis and scientific graphing were performed using IBM SPSS22.0 Statistics for windows, version 22.0 (IBM Corp., Armonk, NY, USA) and GraphPad Prism 9.0 (GraphPad Software, San Diego, CA, USA). Statistical significance was set at *P* < 0.05.

## Supplementary information

Supplementary manuscript

Supplementary figure 1

Supplementary figure 2

Supplementary figure 3

Supplementary figure 4

Supplementary figure 5

Supplementary figure 6

Supplementary table 1

Supplementary table 2

Supplementary table 3

Supplementary table 4

aj-checklist

## Data Availability

The mass spectrometry proteomics data have been deposited to the ProteomeXchange Consortium (http://proteomecentral.proteomexchange.org) with the dataset identifier PXD034266. The RNA-seq data have been deposited in the GEO database under accession code GSE205385. All data generated or analyzed during this study are included in this article and its supplementary information files.
