# Supplementary material for: CRMP2 derived from cancer associated fibroblasts facilitates progression of ovarian cancer via HIF-1α-glycolysis signaling pathway
Source: Cell Death Dis. 2022 Aug 4;13(8):675. doi: 10.1038/s41419-022-05129-5 (PMC9352901; doi:10.1038/s41419-022-05129-5)
Supplement: Supplementary file 1 — Supplementary manuscript [file 41419_2022_5129_MOESM1_ESM.docx]

**Supplementary material and methods**

**Isolation of CAFs and NOFs**

Primary CAFs and NOFs were isolated from EOC tissues and their matched para-carcinoma tissues of three EOC patients who underwent surgery during 2019 and 2020 at Obstetrics and Gynecology Hospital of Shanghai, Fudan University. Patients who received any preoperative chemotherapy or radiotherapy were excluded. Tissues were washed with phosphate-buffered saline (PBS) to remove unwanted tissues (fat, necrotic material, and blood coagula). Then, tissues were minced into small pieces (1–2 mm^3^) and digested in the mixed digestion solution containing 0.25% trypsin solution (Gibco, Gaithwesburg, MD, USA), 2 mg/ml collagenase-type IV (Sigma, St Louis, MO, USA) and 1% penicillin/streptomycin (Thermo Fisher Scientific, Waltham, MA, USA) for approximately 2 h on an orbital shaker at 37 °C. Then, the cell suspension was centrifuged at 1000 rpm for 10 min after filtration though 75 um pore filter. Primer cells were cultured in DMEM/F12 (HyClone, UT, USA) with 10% FBS at 37 °C in humidified 5% CO_2_, and the medium was changed after 48 h.

**Cell immunofluorescence**

Cells were seeded in a 12-well plate with coverslips in each well. After PBS washing, cells were fixed with 4% paraformaldehyde and permeabilized with 0.1% Triton X-100 for 10 min. Cells were then blocked with 1% bovine serum albumin (BSA) for 1 h at room temperature before incubation with primary antibodies against anti-FAP (1:100, CST, Boston, MA, USA), anti-α-SMA (1:100, ABclonal, Wuhan, China) and anti-CRMP2 (1:100, ABclonal) at 4°C overnight. FITC (green) or red (red)-conjugated secondary antibodies (Santa Cruz, CA, USA) were incubated with primary antibodies for 1 h. Nuclear staining was performed with DAPI and photographed using fluorescence microscope.

**Liquid chromatography mass spectrum (LC-MS/MS) analysis**

The samples were resuspended in a lysis buffer containing phosphatase inhibitor cocktail and PMSF. After an in-solution digestion procedure, protein peptides were lyophilized at 30°C and stored at -80°C. Proteome analysis was performed using a Fusion mass spectrometer (Thermo Fisher scientific, USA). The dried peptides were dissolved in 2% acetonitrile with 0.1% formic acid and loaded on a reverse-phase analytical column (Eksigent, C18, 3μm, 150mm×75μm). Eluted peptides were separated over a 78 min gradient of phase B (98% CAN with 0.1% formic acid) at 300 nL/min flow rate. An electrospray voltage of 2.0 kV versus inlet of mass spectrometer was used.

**RNA sequencing and bioinformatics analysis**

Total RNA was isolated from cells using an RNA-Quick purification kit (ES science, China) according to the manufacturer’s protocol. Briefly, the mRNA was enriched by oligodT according to the instructions of NEBNext Poly(A) mRNA Magnetic Isolation Module (NEB, USA). The fragmented were fragmented to approximately 200bp. The RNA fragments were subjected to first and second strands cDNA synthesis, followed by adaptor ligation and low cycle enrichment according NEBNext Ultra RNA Library Prep Kit for Illumina instruction. Purified library products were evaluated using Agilent 2200 TapeStation and Qubit (Thermo Fisher Scientific, USA). The libraries were sequenced by Illumina (Illumina, USA) with paired-end 150 bp at RiboBio Co. Ltd (RiboBio, China).

For bioinformatic analysis, sequencing read quality was inspected using the FastQC software. For gene differential expression analysis, statistically significant DE genes were obtained by an adjusted P < 0.05, and |log2(foldchange)| > 1 using the DEseq/DESeq2/edgeR/DEGseq software. Finally, a hierarchical clustering analysis was performed using the R language package, gplots, according to the RPKM values.

GO terms and KEGG pathway enrichment analyses were performed using “clusterProfiler” package in the RBioconductor /KOBAS3.0 software. Co-expression network was constructed by calculating Pearson correlation coefficient and P-value between multiple genes and illustrated using Cytoscape software. Interaction network of protein-protein internet was constructed using the STRING database and visualised using Cytoscape software.

**Cell co-culture models**

The co-cultured models were conducted using 6-well and 0.4 um Transwell inserts (Corning, Acton, MA, USA). SKOV3 and A2780 cells with a density of 1×10^6^ per well were seeded in the lower chamber, while 2×10^5^ CAFs were seeded in the upper chamber. The culture mediums used in co-culture model were the same as previous described. After co-culturing for appropriate time, OCCs were then collected for the extraction of total protein and immunoblot.

**Human umbilical vein endothelial cells (HUVECs) tubule formation assay.**

Growth factor-reduced Matrigel (BD, CA, USA) was thawed overnight. A total of 100 μl Matrigel was added to each well of pre-cooled 96-well plates and incubated at 37°C cell incubator for 1 h. HUVECs were starved for 24 h before seeding on the pre-coated wells. HUVECs (3×10^4^ per well) were seeded on solid Matrigel with 100 μl of different groups of culture medium. The capillary tubes were observed at different time points within 6 h under a 100×bright-field microscope. The total branch nodes and completed tubes were quantified using ImageJ software (Rawak Software, Germany). Three independent experiments were performed for each treatment group.

**Supplementary figure legends**

**Fig. 1** (A) Immunofluorescence staining for α-SMA and FAP of CAFs and NOFs. (B) Gene Ontology (Go) analysis displayed the top 10 sub-cellular locations of differential proteins. (C, D and E) The 10 major biological functional processes, molecular functions and KEGG signaling pathways of differential expressed proteins.

**Fig. 2** (A) ELISA assay was used to detect concentration of CRMP2 in the supernatant of CAFs, NOFs, and CAFs treated with IgG and CRMP2 neutralization antibody (Ab). (B) A2780 cells were cultured with CAF-CM, NOF-CM, CAF-CM-IgG, and CAF-CM-CRMP2-Ab respectively. Wound healing assay was performed to measure cell migration ability at the time points 0-h, 24-h and 48-h. (C) Cell mobility was examined by wound healing when A2780 cells were treated with different concentration of recombination human CRMP2 (r-CRMP2). (D) RT-qPCR detected the background expression of CRMP2 in SKOV3 and A2780. (E) The transfection efficacy of over-expressed CRMP2 plasmids in A2780 was verified by RT-qPCR. (F and G) RT-qPCR and western blot detected the silencing efficacy of four targeted si-RNAs of CRMP2 in primary CAFs. (H) RT-qPCR validated the silencing effect of CRMP2-si-RNA3 (KD-3) and CRMP2-si-RNA4 (KD4) in SKOV3 cells. Results are presented as the mean ± SD of three independent experiments. ***P<0.001.

**Fig. 3** (A-E) Silencing CRMP2 in SKOV3 cells with CRMP2-si-RNA3 (KD3) and CRMP2-si-RNA4 (KD4). (A) Cell viability was detected by CCK8 assay in SKOV3-NC, SKOV3-KD3 and SKOV3-KD4 cells. (B and C) Cell migration ability was measured by wound healing assay (100× magnification). (D and E) Tanswell assays were used to observe cell invasion ability (100× magnification). (F-J) Overexpressing CRMP2 in A2780 cells using DPYSL2(CRMP2)-pTSB02-GFP-PURO plasmid. (F) CCK8 assay detected cell viability in CRMP2 over-expressed A2780 (A2780-OE) and A2780-Vector. (G and H) Cell migration ability was observed via wound healing assay (100× magnification). (I and J) Transwell assay was used to assess cell invasion ability of A2780-OE and A2780-Vector (100× magnification). Results are presented as the mean ± SD of three independent experiments. **P<0.01, ***P<0.001, ns: not significant.

**Fig. 4** (A) Volcano plot presented the up-regulated genes (red spots) and the down-regulated genes (green spots) in A2780 treated with r-CRMP2 group (|log2[fold change]|)>1 and Q-value<0.05). (B) GO functional annotation analysis presents the biological process (BP), cellular component (CC) and molecular function (MF) of differential genes. Responding to hypoxia and glycolysis were the predominant BP of the differential genes (GOBPID:00016666, P=4.73e-06). (C) GEPIA public database revealed that CRMP2 was positively correlated HIF-1α. (R=0.46 and P=0). (D and E) Western blotting validated PI3K-Akt-mTOR pathway using co-culture models from one day (1D) to three days (3D). UN1-3: cells untreated with CAF-CM were set as the controls of 1-3 day. (F and G) Western blotting detected the PI3K-Akt-mTOR pathway after treatment with different concentrations of r-CRMP2(ng/ml).

**Fig. 5** (A and B) The full-length HIF-1α western blots of co-culture model. UN1-3: cells untreated with CAF-CM were set as the controls of 1-3 day. (C and D) The full-length HIF-1α western blots under treatment of different concentrations of r-CRMP2 (0,10,20,40ng/ml). (E and F) The full-length HIF-1α western blots after knocking down CRMP2 in SKOV3 cells and overexpressing CRMP2 in A2780 cells. UN: untreated.

**Fig. 6** (A) HUVECs were cultured with CAF-CM, NOF-CM, CAF-CM +IgG, and CRMP2-neutralization antibody (Ab). Representative tube formation images were photographed at the time point of 6-h after HUVECs were cultured on the BD Matrigel. The number of branch nodes and loops were analyzed by ImageJ software. (B) Tube formation assay was performed to observe tube formation ability of HUVECs cultured with different concentration of r-CRMP2. (C and D) Immunofluorescence (IF) analysis validated α-SMA and CRMP2 expression in ovarian cancer tissues (T) and in para-carcinoma tissues (P). Results are presented as the mean ± SD of three independent experiments. *P < 0.05, ***P < 0.001.
