# Supplementary figures and images for: CRMP2 derived from cancer associated fibroblasts facilitates progression of ovarian cancer via HIF-1α-glycolysis signaling pathway
Source: Cell Death Dis. 2022 Aug 4;13(8):675. doi: 10.1038/s41419-022-05129-5 (PMC9352901; doi:10.1038/s41419-022-05129-5)

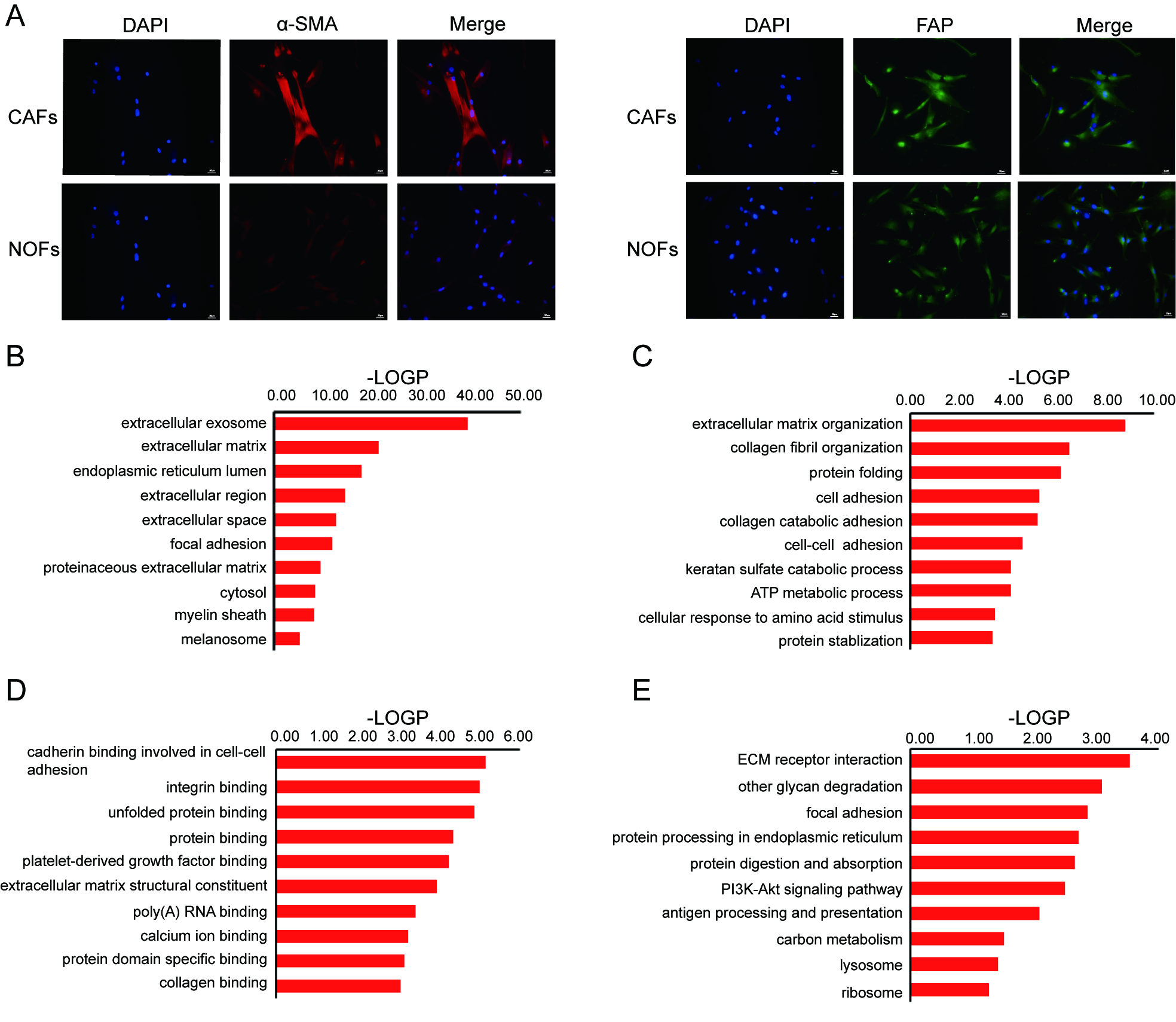

Supplement: Supplementary file 2 — Supplementary figure 1 [file 41419_2022_5129_MOESM2_ESM.tif]

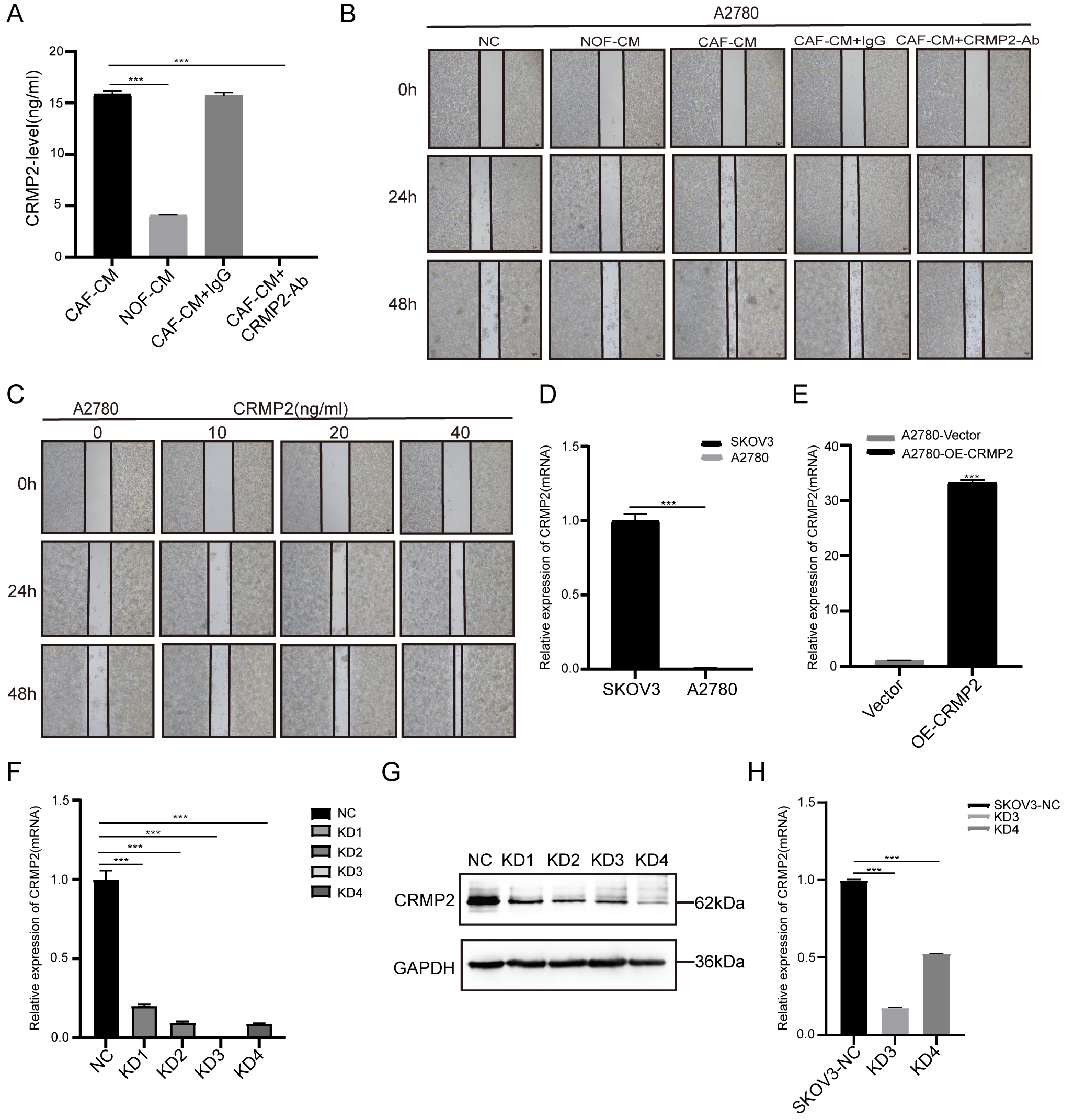

Supplement: Supplementary file 3 — Supplementary figure 2 [file 41419_2022_5129_MOESM3_ESM.tif]

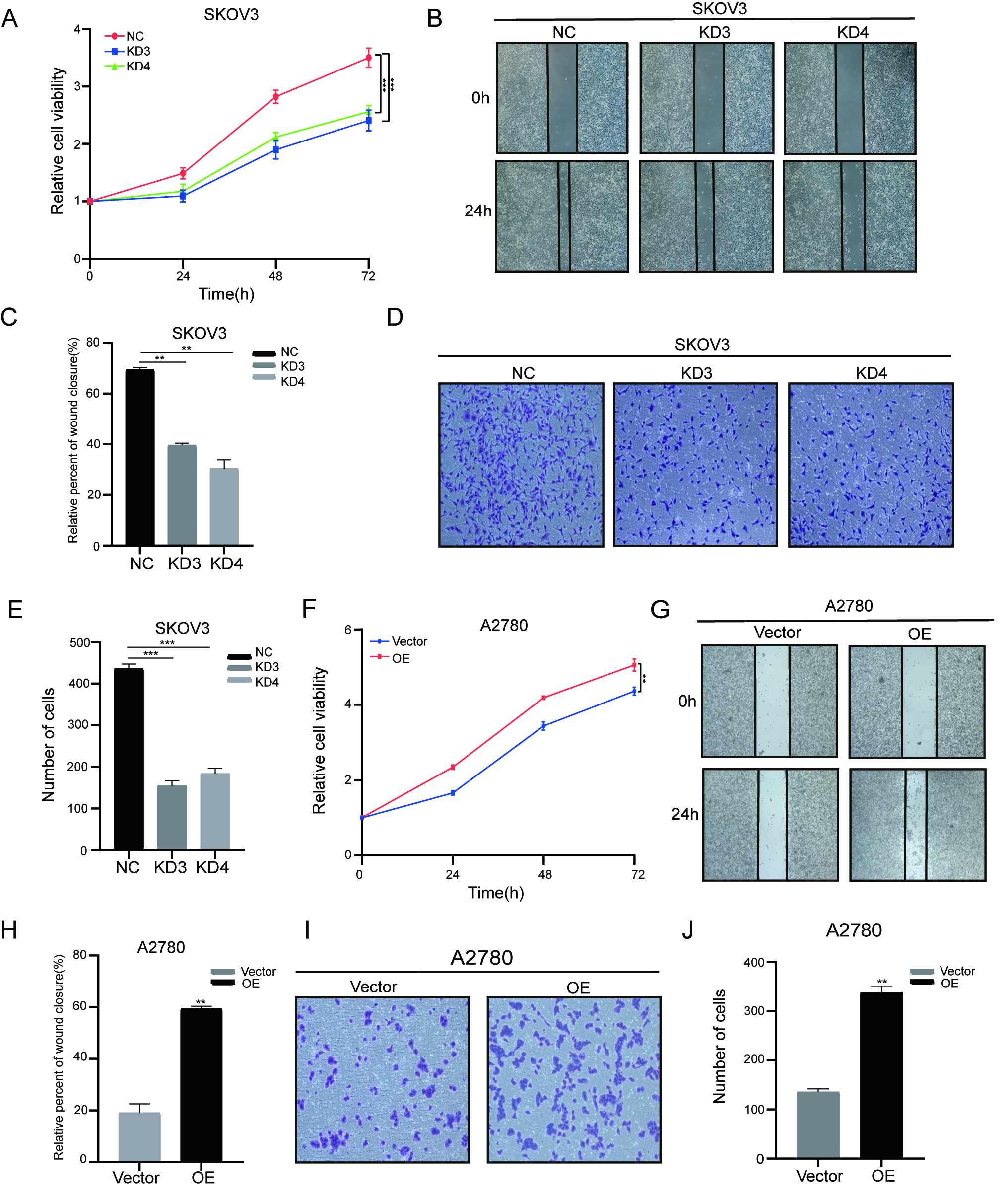

Supplement: Supplementary file 4 — Supplementary figure 3 [file 41419_2022_5129_MOESM4_ESM.tif]

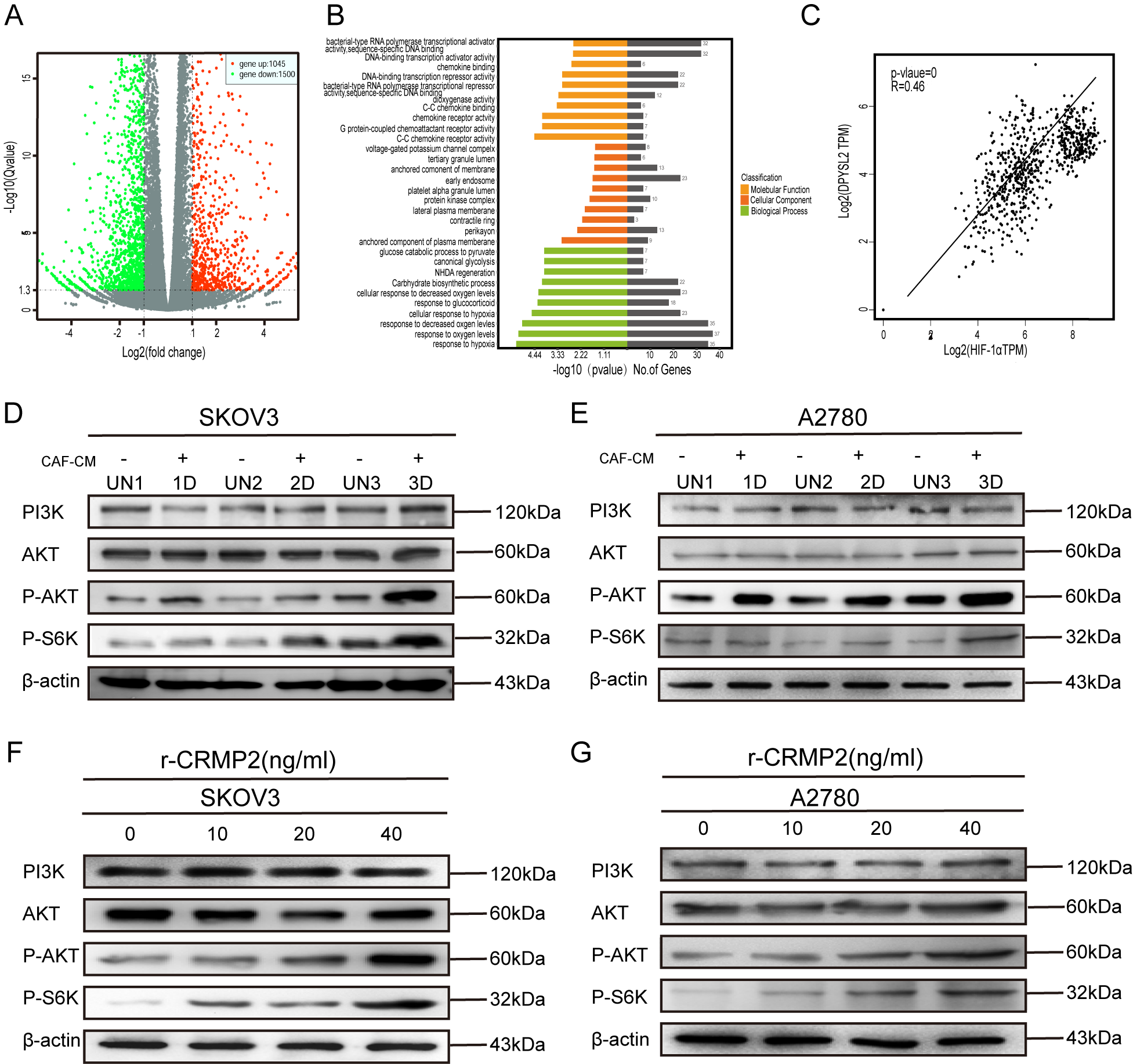

Supplement: Supplementary file 5 — Supplementary figure 4 [file 41419_2022_5129_MOESM5_ESM.tif]

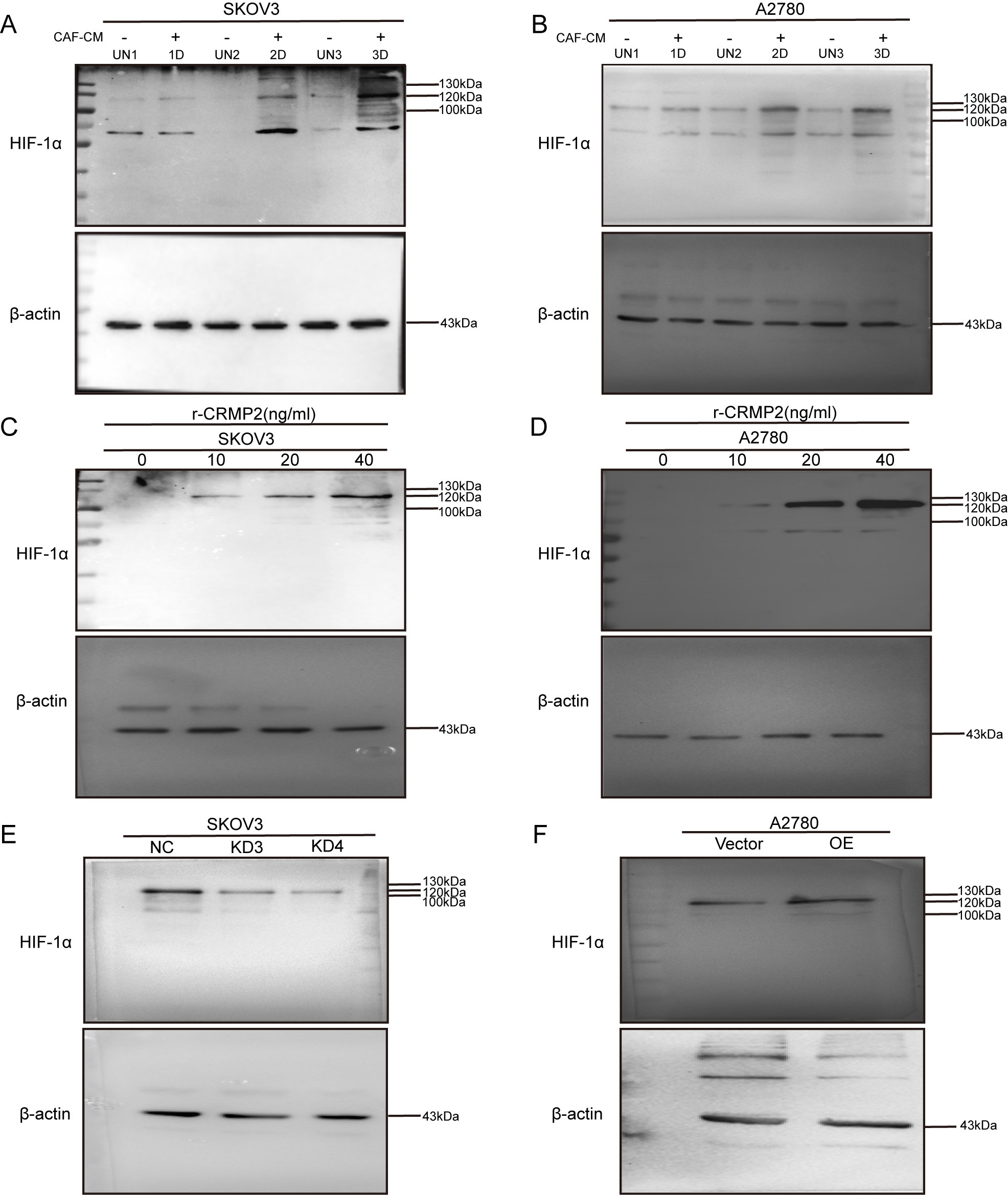

Supplement: Supplementary file 6 — Supplementary figure 5 [file 41419_2022_5129_MOESM6_ESM.tif]

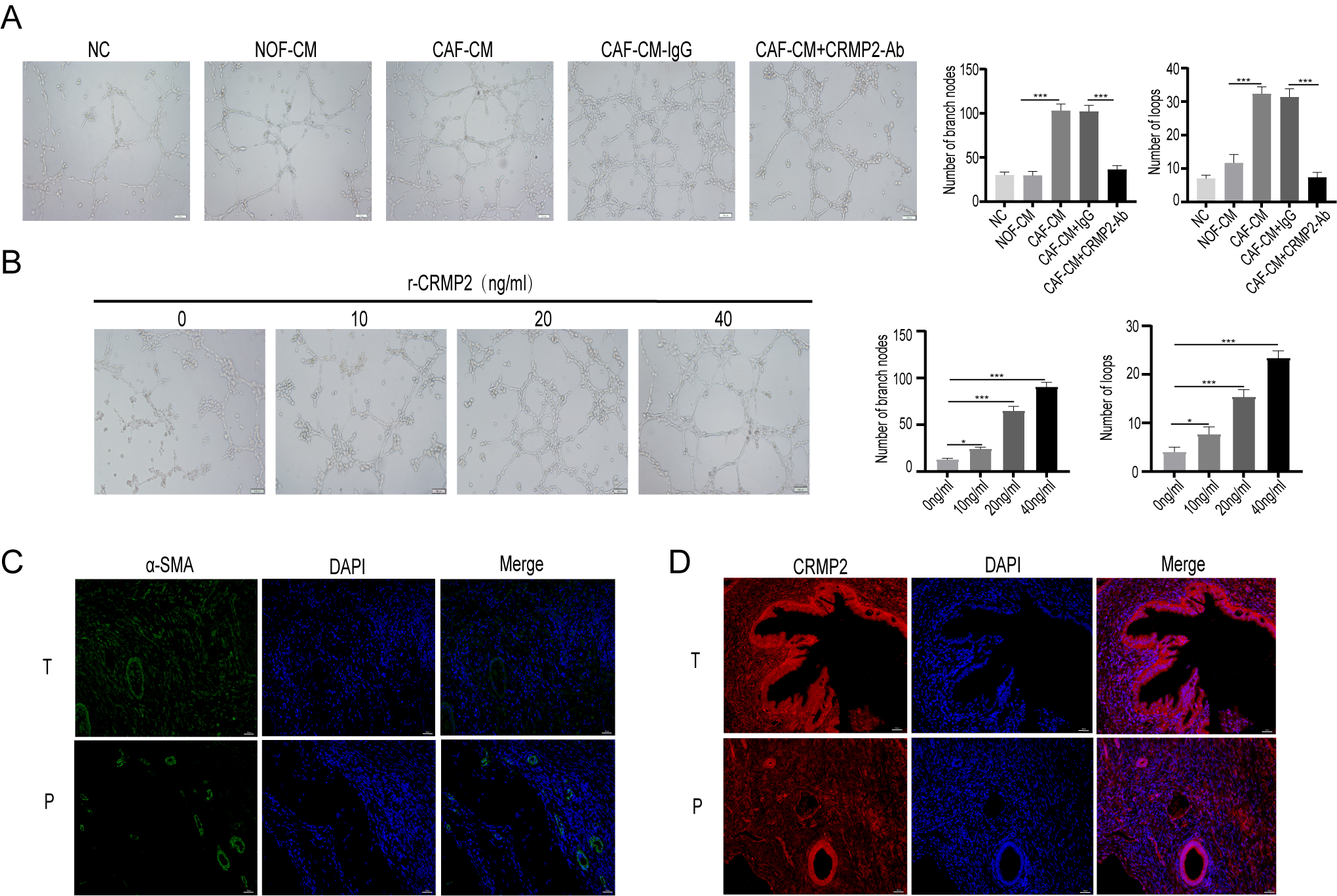

Supplement: Supplementary file 7 — Supplementary figure 6 [file 41419_2022_5129_MOESM7_ESM.tif]
