# Supplementary material for: CRMP2 derived from cancer associated fibroblasts facilitates progression of ovarian cancer via HIF-1α-glycolysis signaling pathway
Source: Cell Death Dis. 2022 Aug 4;13(8):675. doi: 10.1038/s41419-022-05129-5 (PMC9352901; doi:10.1038/s41419-022-05129-5)
Supplement: Supplementary file 8 — Supplementary table 1 [file 41419_2022_5129_MOESM8_ESM.docx]

| Accession number | Entry name | Protein name | Gene name | URL | Ratio | P value |
| --- | --- | --- | --- | --- | --- | --- |
| O95965 | ITGBL_HUMAN | Integrin beta-like protein 1 | ITGBL1 OSCP TIED | https://www.uniprot.org/uniprot/O95965 | 6.47 | 0.002 |
| Q16555 | DPYL2_HUMAN | Dihydropyrimidinase-related protein 2 (DRP-2) (Collapsin response mediator protein-2) (CRMP2) | DPYSL2 CRMP2 ULIP2 | https://www.uniprot.org/uniprot/Q16555 | 5.44 | 0.007 |
| Q07092 | COGA1_HUMAN | Collagen alpha-1 (XVI) chain | COL16A1 FP1572 | https://www.uniprot.org/uniprot/Q07092 | 3.59 | 7.90E-06 |
| P26639 | SYTC_HUMAN | Threonine--tRNA ligase 1, cytoplasmic (Threonyl-tRNA synthetase) (ThrRS) (Threonyl-tRNA synthetase 1) | TARS1 TARS | https://www.uniprot.org/uniprot/P26639 | 3.42 | 0.009 |
| Q15063 | POSTN_HUMAN | Periostin (PN) (Osteoblast-specific factor 2) (OSF-2) | POSTN OSF2 | https://www.uniprot.org/uniprot/Q15063 | 3.35 | 0.031 |
| Q04917 | 1433F_HUMAN | 14-3-3 protein eta (Protein AS1) | YWHAH YWHA1 | https://www.uniprot.org/uniprot/Q04917 | 3.15 | 0.021 |
| Q99538 | LGMN_HUMAN | Legumain (Asparaginyl endopeptidase) (Protease, cysteine 1) | LGMN PRSC1 | https://www.uniprot.org/uniprot/Q99538 | 3.08 | 0.003 |
| Q99497 | PARK7_HUMAN | Parkinson disease protein 7 (Maillard deglycase) | PARK7 | https://www.uniprot.org/uniprot/Q99497 | 3.00 | 0.007 |

**Supplementary table 1. The top eight differential proteins expressed in the supernatant of CAFs compared with NOFs.**
