# Supplementary material for: CRMP2 derived from cancer associated fibroblasts facilitates progression of ovarian cancer via HIF-1α-glycolysis signaling pathway
Source: Cell Death Dis. 2022 Aug 4;13(8):675. doi: 10.1038/s41419-022-05129-5 (PMC9352901; doi:10.1038/s41419-022-05129-5)
Supplement: Supplementary file 9 — Supplementary table 2 [file 41419_2022_5129_MOESM9_ESM.docx]

**Supplementary table 2. The antibodies and dilution rate used in this research.**

| Name of Antibody | Company | Code | Dilution ratio |
| --- | --- | --- | --- |
| FAP | CST | #66562 | 1:1000 |
| α-SMA | ABclonal | A1011 | 1:500 |
| Vimentin | Arigo | ART66199 | 1:1000 |
| CRMP2 | ABclonal | A14570 | 1:500 |
| HIF-1α | Abcam | ab51608 | 1:1000 |
| HK2 | ABclonal | A0994 | 1:500 |
| PGK1 | Santa Cruz | Sc-130335 | 1:1000 |
| PFKFB3 | ABclonal | A3934 | 1:500 |
| PKM2 | Santa Cruz | sc-365684 | 1:500 |
| PDK1 | ABclonal | A8930 | 1:1000 |
| LDHA | ABclonal | A0861 | 1:1000 |
| GLUT3 | ABclonal | A4137 | 1:500 |
| VEGFA | Santa Cruz | sc-271789 | 1:1000 |
| PI3K kinase p110α | CST | #4225 | 1:1000 |
| AKT | CST | #9272 | 1:1000 |
| Phospho-AKT(Ser473)  Phospho-S6K(Ser235/236) | CST  CST | #4060  #4858 | 1:1000  1:1000 |
| GADPH | ABclonal | A19056 | 1:2000 |
| β-actin | Proteintech | 20536-1-AP | 1:2000 |
| goat antirabbit IgG  goat antimouse IgG | ABclonal  Jackson | AS014  115-035-003 | 1:4000  1:4000 |
