# Supplementary material for: CRMP2 derived from cancer associated fibroblasts facilitates progression of ovarian cancer via HIF-1α-glycolysis signaling pathway
Source: Cell Death Dis. 2022 Aug 4;13(8):675. doi: 10.1038/s41419-022-05129-5 (PMC9352901; doi:10.1038/s41419-022-05129-5)
Supplement: Supplementary file 10 — Supplementary table 3 [file 41419_2022_5129_MOESM10_ESM.docx]

**Supplementary table 3. A list of primers used in this study.**

| Gene | Forward sequence (5’ to 3’) | Reverse sequence (5’ to 3’) |
| --- | --- | --- |
| β-actin | CTACCTCATGAAGATCCTCACC | AGTTGAAGGTAGTTTCGTGGAT |
| α-SMA | CTATGAGGGCTATGCCTTGCC | GCTCAGCAGTAGTAACGAAGGA |
| FAP | ATGAGCTTCCTCGTCCAATTCA | AGACCACCAGAGAGCATATTTTG |
| Vimentin | GACGCCATCAACACCGAGTT | CTTTGTCGTTGGTTAGCTGGT |
| CRMP2 | GATCCCCGGAGGAATTGACG | GGCTCAGGAACAACGTGGTC |
| HIF-1α | GAACGTCGAAAAGAAAAGTCTCG | CCTTATCAAGATGCGAACTCACA |
| HK2  PGK1 | GAGCCACCACTCACCCTACT  TGGACGTTAAAGGGAAGCGG | CCAGGCATTCGGCAATGTG  GCTCATAAGGACTACCGACTTGG |
| PFBFB3 | ATTGCGGTTTTCGATGCCAC | GCCACAACTGTAGGGTCGT |
| PKM2 | ATGTCGAAGCCCCATAGTGAA | TGGGTGGTGAATCAATGTCCA |
| PDK1 | CTGTGATACGGATCAGAAACCG | TCCACCAAACAATAAAGAGTGCT |
| LDHA | ATGGCAACTCTAAAGGATCAGC | CCAACCCCAACAACTGTAATCT |
| GLUT3 | GCTGGGCATCGTTGTTGGA | GCACTTTGTAGGATAGCAGGAAG |
| VEGFA | AGGGCAGAATCATCACGAAGT | AGGGTCTCGATTGGATGGCA |
