# Supplementary material for: CRMP2 derived from cancer associated fibroblasts facilitates progression of ovarian cancer via HIF-1α-glycolysis signaling pathway
Source: Cell Death Dis. 2022 Aug 4;13(8):675. doi: 10.1038/s41419-022-05129-5 (PMC9352901; doi:10.1038/s41419-022-05129-5)
Supplement: Supplementary file 11 — Supplementary table 4 [file 41419_2022_5129_MOESM11_ESM.docx]

**Supplementary table 4. si-RNAs used in this study.**

| si-RNA | Forward sequence (5’ to 3’) | Reverse sequence (5’ to 3’) |
| --- | --- | --- |
| DPYSL2-1 | CUGAGUGUGAUCCGGGAUAdTdT | UAUCCCGGAUCACACUCAGdTdT |
| DPYSL2-2 | GAGUGUGAUCCGGGAUAUUdTdT | AAUAUCCCGGAUCACACUCdTdT |
| DPYSL2-3 | CUAACGGAUUGCCAGAUUUdTdT | AAAUCUGGCAAUCCGUUAGdTdT |
| DPYSL2-4 | GACCAACUGCCCGCUGUAUdTdT | AUACAGCGGGCAGUUGGUCdTdT |
